# Intraoperative margin assessment in colorectal cancer via AI-enhanced multiphoton microscopy

**DOI:** 10.1117/1.JBO.31.10.106501

**Published:** 2026-10-09

**Authors:** Qiaoyu Lai, Zilu Lin, Gangqin Xi, Jiajia He, Guangxing Wang, Jun Zhang, Shuangmu Zhuo, Jian Sui

**Affiliations:** a Jimei University, School of Science, Xiamen, China; b Ningbo University of Technology, School of Electronic and Information Engineering, Ningbo, China; c Fujian Provincial Hospital, Fuzhou University Affiliated Provincial Hospital, Department of Gastrointestinal Surgery, Fuzhou, China

**Keywords:** colorectal cancer, margin assessment, multiphoton microscopy, microarchitecture, pyramid vision transformer, feature integration

## Abstract

**Significance:**

Identifying residual tumors at the resection margins is crucial for individuals undergoing colorectal cancer (CRC) surgery. Accurate margin assessment can help minimize residual tumor while avoiding unnecessary removal of healthy tissue.

**Aim:**

This study uses label-free multiphoton microscopy to visualize collagen architecture and develops a feature-enhanced deep-learning model to distinguish CRC from margin-adjacent normal tissue.

**Approach:**

Margin-adjacent normal and carcinoma tissues were imaged using multiphoton microscopy, and 142 quantitative collagen features were extracted from SHG images. We propose PVT-Enhanced, a feature-fusion model that integrates handcrafted collagen features with deep image features derived from PVTv2-b5 through an attention-based fusion strategy. Its performance was compared with LASSO-LR, three convolutional neural networks, and image-only PVTv2-b5.

**Results:**

SHG imaging showed relatively coherent fiber orientation, longer and more continuous collagen fibers, and higher fiber density in margin-adjacent normal tissue, whereas carcinoma tissue showed more disorganized orientation, shorter and more fragmented fibers, and lower fiber density. The PVT-Enhanced model achieved an accuracy of 86.9%, an AUC of 0.923, and a specificity of 96.4%. Only 5 of 137 margin-adjacent normal images were misclassified as CRC.

**Conclusions:**

Our findings suggest that the PVT-Enhanced framework combined with SHG imaging allows accurate, rapid, and label-free tissue characterization that may support intraoperative margin assessment in CRC surgery.

## 1 Introduction

Colorectal cancer (CRC) is one of the most prevalent causes of cancer-related deaths worldwide.^1^^,^^2^ Therefore, there is an urgent need for transformative diagnostic technologies to resolve this issue. Despite advances in clinical practice, early detection of this disease remains challenging. Moreover, precise tumor resection remains challenging because rapid and accurate intraoperative assessment of resection margins is still limited.^3^ Methodologies, such as colonoscopy, biopsy, and cross-sectional imaging, have limitations in the detection of early lesions and microinvasion, as well as in margin delineation.^4^ Histopathological examination of biopsy specimens remains the standard for diagnosis but is invasive, subject to sampling limitations, and requires tissue processing.^5^ Intraoperative frozen-section assessment can provide information on resection margins, but tissue processing is time-consuming, and the resulting information may sometimes be unclear, limiting rapid intraoperative decision-making.^6^ Approximately one-third of patients with CRC develop metastatic disease at presentation or during follow-up, and the 5-year relative overall survival for metastatic CRC remains ∼15%.^7^

Collagen architecture in histologically negative margin-adjacent tissue remains insufficiently characterized. Although magnetic resonance imaging provides important macroscopic information, it is not able to interrogate the tumor microenvironment at the necessary resolution.^8^ Lesion spots can be visualized using optical coherence tomography (OCT). However, the resolution may be insufficient to visualize individual collagen fibers.^9^ Notably, before cellular involvement in the margins, a remodeling effect occurs within the collagen architecture.^10^ In this study, label-free multiphoton microscopy (MPM) was used to visualize the second-harmonic generation (SHG) signal of collagen. SHG imaging provides sub-micron-resolution visualization of collagen architecture, enabling quantitative assessment of fiber continuity, orientation, and density.^11^^,^^12^ MPM also has the potential for *in vivo* tissue imaging because of its penetration depth and relatively low phototoxicity. As an initial proof of concept for such applications, the present study evaluated the proposed approach using unstained fresh-frozen tissue sections rather than intact living tissue. Artificial intelligence (AI) can further assist in extracting and integrating quantitative imaging information.^13^ However, AI-based pathology models require careful validation because limited generalizability and interpretability may affect their clinical application.^14^^,^^15^

Against this background, this study combines quantitative SHG imaging with an optical–AI framework for tissue classification. The goals of this study were (i) to develop quantitative SHG-derived collagen features for differentiating CRC from margin-adjacent normal tissue and (ii) to develop a hybrid AI model integrating quantitative collagen features with deep image features for tissue classification. By combining explicit quantitative collagen descriptors with learned image representations, the proposed framework aims to improve tissue discrimination while retaining biologically relevant structural information. This approach may support more precise intraoperative margin assessment and surgical decision-making, although its clinical benefits require validation in larger patient cohorts.

## 2 Materials and Methods

### 2.1 Sample Preparation

This study included fresh-frozen tissue sections from 10 patients undergoing surgical resection for CRC (eight cases of colon cancer and two cases of rectal cancer). Following resection, the specimens were oriented. The proximal resection margin (PRM) and distal resection margin (DRM) were then marked according to routine pathological procedures. Tissue sampling was designed to include both margin-adjacent normal tissue and carcinoma tissue for comparative analysis. Margin-adjacent normal tissue was defined as the normal mucosa and submucosal layer within 1 cm of the negative resection margin. Histological examination confirmed the absence of tumor infiltration in the specimen. Samples of the carcinoma region were collected from the area of the tumor at least 3 cm away from the negative resection margins. These two tissue categories were compared to characterize collagen differences relevant to the development of margin-assessment methods.

For histopathological assessment and MPM imaging, 10-μm frozen sections were prepared. H&E-stained sections were used for histopathological assessment, while the corresponding unstained sections were used for label-free MPM imaging. Small amounts of phosphate-buffered saline (PBS) were used to avoid tissue dehydration during imaging. An experienced pathologist first reviewed the H&E sections and designated the tumor or normal tissue areas as regions of interest (ROIs). After annotation by the pathologist, these ROIs were imaged using MPM on the corresponding unstained sections to simultaneously collect both two-photon-excited fluorescence (TPEF) and SHG signals.

### 2.2 Multiphoton Microscopy

The MPM imaging experiment was performed using a high-throughput inverted scanning microscope (DMi8, Leica, Germany) with an InSight X3 femtosecond laser (Spectra-Physics, California United States). The laser parameters were as follows: pulse width of 120 fs, repetition rate of 80±0.5  MHz, and excitation wavelengths ranging from 680 to 1300 nm. The detection on the back scanner employed a water-immersion 25x/0.95 NA objective, which offers high signal collection.^16^ In the dual-channel detection pathway, a dichroic mirror was used to spectrally separate the photoexcited emission into SHG and TPEF channels. All experiments were conducted using an excitation wavelength of 810 nm. The recorded SHG signal was within the 395 to 415 nm spectral range. The TPEF signal was detected in the wavelength range of 430 to 650 nm.

### 2.3 Dataset Partitioning and ROI Selection

Data partitioning was performed at the patient level. Patients P01–P07 were assigned to the training cohort and P08–P10 to the held-out validation cohort. All tissue sections, ROIs, and image patches from the same patient were retained within the same partition, and no image-level random splitting was used to create the training and validation cohorts.

The final dataset included 50 tissue sections from 10 patients. For each section, 1 to 3 spatially nonoverlapping ROIs were selected with reference to the corresponding H&E-stained section. Depending on ROI size, multiple MPM fields were acquired at a resolution of 1024×1024  pixels. Multiple 512×512-pixel SHG image patches were subsequently cropped from these fields for analysis, with no spatial overlap among the retained patches. SHG images were used for quantitative collagen feature extraction and all machine-learning and deep-learning analyses. TPEF and merged SHG/TPEF images were used for tissue visualization, whereas H&E-stained sections served as histopathological references for ROI annotation and were not used as model inputs. In total, 76 ROIs yielded 1268 SHG image patches, including 879 in the training cohort and 389 in the held-out validation cohort. Patient-level details are provided in Table 1.

**Table 1:** Patient-level distribution of tissue sections, ROIs, and SHG image patches in the training and validation cohorts.

Patient ID	Anatomical site	Data partition	Tissue categories	Tissue sections, n			ROIs, n			Image patches, n		
				CRC	Margin-adjacent normal	Total	CRC	Margin-adjacent normal	Total	CRC	Margin-adjacent normal	Total
P01	Rectum	Training	CRC only	1	0	1	1	0	1	13	0	13
P02	Colon	Training	Both	2	2	4	5	3	8	80	50	130
P03	Colon	Training	Both	2	2	4	4	4	8	48	65	113
P04	Colon	Training	Both	2	5	7	2	5	7	25	98	123
P05	Colon	Training	CRC only	3	0	3	7	0	7	105	0	105
P06	Colon	Training	Both	2	2	4	6	4	10	102	55	157
P07	Colon	Training	Margin-adjacent normal only	0	4	4	0	11	11	0	238	238
P08	Rectum	Validation	Both	4	5	9	4	5	9	22	14	36
P09	Colon	Validation	Both	5	3	8	5	3	8	144	41	185
P10	Colon	Validation	Both	2	4	6	3	4	7	86	82	168
**Total**			**10 patients**	**23**	**27**	**50**	**37**	**39**	**76**	**625**	**643**	**1268**

CRC, colorectal cancer; MPM, multiphoton microscopy; ROI, region of interest.

Training cohort, n=7 patients; validation cohort, n=3 patients. Patient identifiers are anonymized. Not all patients contributed both CRC and margin-adjacent normal tissue images.

### 2.4 Collagen Feature Extraction

We obtained information on the special features of the collagen fibers from the SHG images using MATLAB (R2017b). A total of 142 handcrafted features were acquired. Here, handcrafted features refer to predefined quantitative collagen descriptors extracted from SHG images using custom image-analysis procedures, comprising morphological, histogram, gray-level co-occurrence matrix (GLCM), and Gabor features. Eight morphological features were assessed: collagen percentage area, fiber density, average fiber length, average fiber width, average fiber straightness, crosslink density, average crosslink spacing, and fiber orientation. The remaining features comprised six histogram-based features, 80 GLCM-based features, and 48 Gabor wavelet transform features.

To reduce feature dimensionality, the least absolute shrinkage and selection operator (LASSO) was applied, which imposes an L1 penalty on the regression coefficients and shrinks some coefficients toward zero.^17^ The feature matrix and binary labels (CRC and margin-adjacent normal) were fed to the glmnet package of the R environment.^18^ All features were standardized using the normalization functions included in the package so that the parameters were comparable.^19^ Five-fold cross-validation was used within the LASSO procedure to select the regularization parameter λ according to the one-standard-error criterion (lambda.1se). This cross-validation step was used for parameter selection rather than for reporting the final model performance. Features with non-zero coefficients under the selected λ were retained as the final discriminative feature subset. We used the selected features to train the logistic regression classifier.^20^^,^^21^ The resulting feature-based model is referred to as LASSO-LR and serves as the feature-only baseline. LASSO feature selection was used only for this baseline model and was not applied to the subsequent deep-learning models. Then, for each sample i, we defined the decision function as follows:^21^

$$z_{i}=\beta_{0}+\overset{k}{\underset{j=1}{\sum }}\beta_{j}X_{ij},$$

where k is the number of retained features and $\beta_{j}$ is the learned coefficient for feature j. Finally, we calculated the probability that a sample was cancerous using the following Sigmoid function:^22^

$$P(y_{i}=1|X_{i})=\frac{1}{1+e^{-z_{i}}}.$$

Thereafter, using the classification threshold, we converted the predicted probability to a discrete label: the sample was classified as CRC tissue when P(y=1|X)≥threshold; the sample was classified as margin-adjacent normal tissue otherwise.

### 2.5 Deep Learning Models

#### 2.5.1 PVTv2-b5 model architecture

The backbone network used in this study was the Pyramid Vision Transformer v2 base-5 (PVTv2-b5) model.^23^ The PVTv2-b5 model was initialized with ImageNet-pretrained weights and subsequently fine-tuned on the SHG training cohort for 50 epochs. PVTv2-b5 is a hierarchical vision transformer designed to incorporate convolutions into the transformer architecture. In particular, convolution-based overlapping patch embedding is used for early feature extraction and spatial down-sampling, whereas long-range modeling is accomplished by transformer blocks (Fig. 1, yellow area). A close analysis reveals that PVTv2-b5 introduces three important architectural components: overlapping patch embedding, spatial-reduction attention using average pooling, and a convolution-based feed-forward network (FFN), as shown in Fig. 1, pink area. PVTv2-b5 was selected because its hierarchical pyramid architecture provides multiscale feature representations, while its self-attention mechanism enables modeling of long-range spatial relationships in collagen organization.

**Fig. 1:**
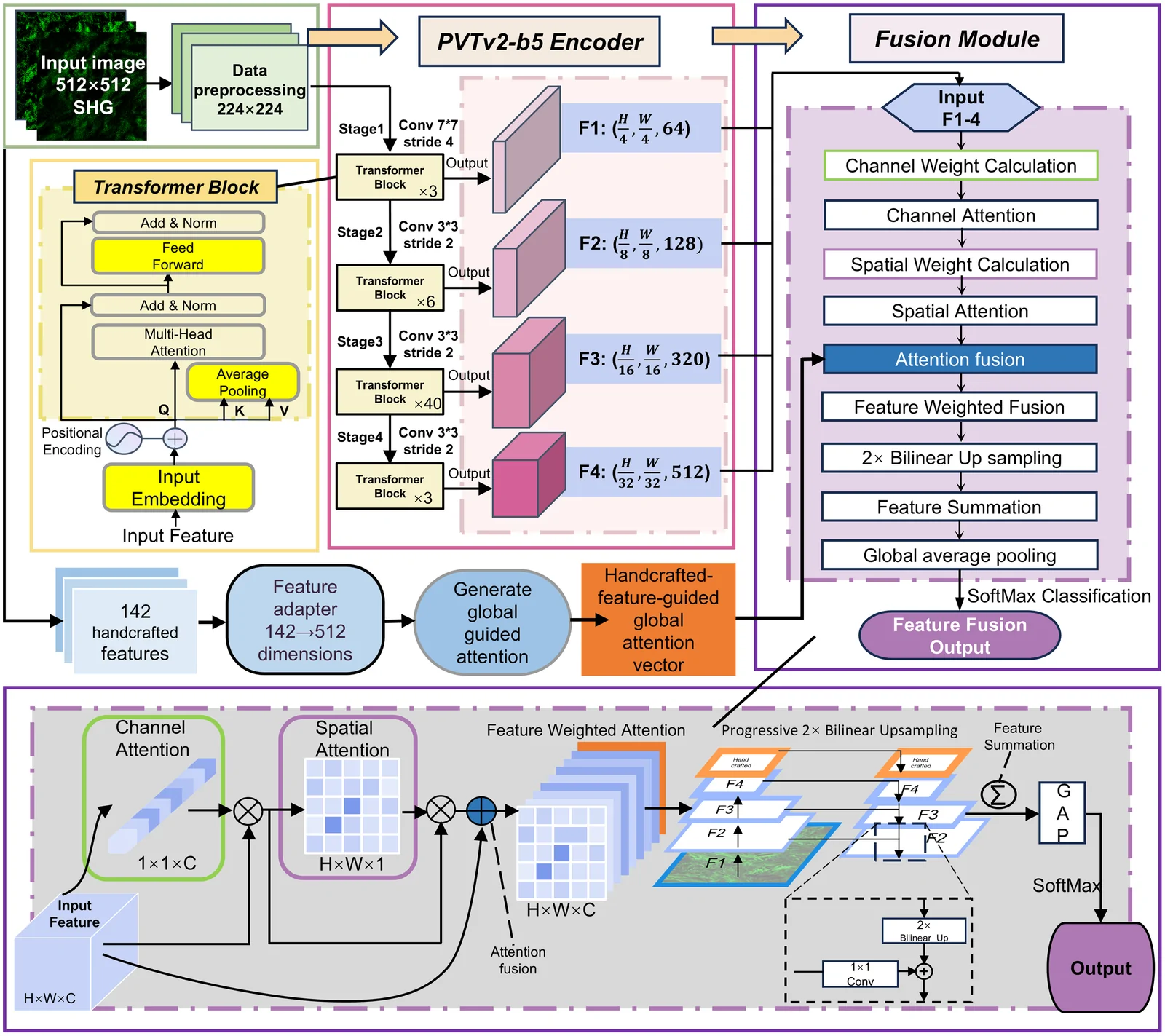
Feature-fusion PVT-Enhanced architecture. Only SHG images were used as image inputs to the classification models.

The model input comprised 512×512 SHG images that were preprocessed and resized to 224×224  pixels. The input images were first fed into the convolution-based overlapping patch embedding layer with a kernel size of 7×7 and a stride of 4 for the initial feature extraction and coarse spatial down-sampling. A four-stage encoder was used to progressively learn hierarchical feature representations. In stages 2, 3, and 4, overlapping patch embedding was performed using 3×3 convolutions with a stride of 2. The four-stage transformer encoder, with depths of 3, 6, 40, and 3, respectively, produced the multiscale feature maps F1, F2, F3, and F4 with spatial resolutions of 56×56, 28×28, 14×14, and 7×7. The corresponding channel dimensions of F1–F4 were 64, 128, 320, and 512, respectively.

The feature fusion module (Fig. 1, purple area) takes the four multi-scale feature maps from the encoder.^24^ These features are then recalibrated using a serial attention mechanism inspired by a sequential channel–spatial attention strategy.^25^ In this module, inter-channel dependencies are first captured with a 1×1 convolution, followed by a ReLU operation to produce a channel weight vector (1×1× C). The global average of the channel-weighted features is used to generate spatial attention, which is reconstructed through a 3×3 convolution to generate a spatial weight matrix (H×W×1), as shown in the gray area of Fig. 1. The combined channel-spatial attention emphasizes key collagen-related information in the multiscale features, thereby enhancing the discriminative quality of the resulting feature maps.

However, progressive down-sampling in deeper transformer stages may lead to the loss of fine microstructural cues that are vital for the identification of tumor invasion fronts and subtle collagen remodeling. To overcome this limitation, we added the 142 handcrafted features described above (morphological, histogram-based, GLCM, and Gabor wavelet features) to provide more fine-scale texture and morphological information,^26^ as shown by the orange block in Fig. 1. The fusion process includes projecting the 142-dimensional handcrafted feature vector into a 512-dimensional representation to match the feature dimension of stage 4 through a fully connected feature adapter. We used layer normalization and ReLU activation to stabilize the data distribution and improve nonlinear representations. Spatial and channel attention mechanisms have been explored for feature recalibration in medical imaging.^27^ Subsequently, a global guided attention vector was generated from the adapted handcrafted feature vector. This vector was combined with the encoder’s deep features (F4) from PVTv2-b5 through an attention-fusion operation to obtain optimized weighted fusion features. To enable multiscale aggregation, F1–F3 were first projected to 512 channels using 1×1 convolutions to match F4. The fused F4 feature was then up-sampled by 2× using bilinear interpolation (align_corners = False) and added element-wise to F3; the resulting feature was successively up-sampled and added to F2 and F1 in the same manner. Element-wise summation was used after spatial and channel alignment to integrate the multiscale features at corresponding spatial locations. Compared with concatenation, this strategy avoids channel expansion and maintains a fixed 512-channel representation without requiring an additional projection step. Finally, the aggregated features were compressed using global average pooling, and the two-class outputs were converted into class probabilities using the SoftMax function, with the final class determined by the maximum probability. The resulting model integrating PVTv2-b5 image features with the 142 handcrafted collagen features is termed PVT-Enhanced.

#### 2.5.2 Traditional CNN architectures

Three representative CNN architectures, VGG-16, ResNet-50, and MobileNet-v2, were evaluated as image-only baselines for the binary classification of CRC and margin-adjacent normal tissue. These models represent conventional deep convolutional, residual, and lightweight network designs, respectively. All three were initialized with ImageNet-pretrained weights and fine-tuned on the SHG training data. ResNet-50 employs residual learning with shortcut connections to facilitate the optimization of deep networks.^28^ VGG-16 uses a deep sequential architecture based on stacked 3×3 convolutional layers for hierarchical image feature extraction.^29^ MobileNet-v2 is a lightweight CNN based on depthwise separable convolutions and inverted residual bottlenecks.^30^ A side-by-side comparison of the evaluated model frameworks, including their model types, inputs, and key architectural characteristics, is provided in Table 2.

**Table 2:** Side-by-side comparison of the evaluated model frameworks.

Model	Type	Input	Key architecture
LASSO-LR	ML	142 handcrafted features	LASSO: 142 → 8 + LR
ResNet-50	CNN	SHG	Residual connections
VGG-16	CNN	SHG	Stacked 3×3 convolutional layers
MobileNet-v2	CNN	SHG	Depthwise separable convolutions + inverted residuals
PVTv2-b5	Transformer	SHG	Four-stage pyramid + overlapping patch embedding + self-attention
**PVT-Enhanced**	Hybrid model	SHG + 142 handcrafted features	Channel–spatial attention + attention-based feature fusion + progressive 2× bilinear upsampling + element-wise summation

LASSO, least absolute shrinkage and selection operator; CNN, convolutional neural network; LR, logistic regression; ML, machine learning; PVT, pyramid vision transformer; SHG, second-harmonic generation. Handcrafted features refer to the 142 collagen features extracted from SHG images (eight morphological and 134 texture features). For LASSO-LR, eight features with nonzero coefficients were retained for LR classification; PVT-Enhanced incorporated all 142 handcrafted features. Architectural details and references are provided in Sec. 2.5.

### 2.6 Performance Evaluation and Statistical Analysis

All models were evaluated on the same held-out validation cohort of 389 SHG images. The reported performance values were derived from this cohort rather than averaged across cross-validation folds or repeated runs. Performance was assessed using AUC, accuracy, sensitivity, specificity, PPV, and F1 score, with 95% confidence intervals. AUC confidence intervals were calculated using DeLong’s method, and AUCs were compared using paired DeLong tests. Wilson intervals were used for accuracy, sensitivity, specificity, and PPV, whereas F1-score confidence intervals were estimated using 10,000 nonparametric bootstrap resamples. Pairwise AUC comparisons with PVT-Enhanced were adjusted for multiple comparisons using the Holm method.

## 3 Results

### 3.1 Label-Free Multiphoton Imaging Reveals Distinct Collagen Architectures at Surgical Margins

We employed label-free MPM to obtain high-resolution images of stromal collagen and cell composition at the surgical margin. An orderly comparison was performed between the tissue adjacent to the inked margin and the tumor mass. Stromal organization underwent distinct changes (Fig. 2). In histologically confirmed margin-adjacent normal tissue within 1 cm of the negative resection margin (Fig. 2, Region 1), SHG imaging revealed closely packed collagen fibers with relatively coherent orientation [Fig. 2(a)]. The magnified ROI showed relatively coherent fiber orientation, longer and more continuous collagen fibers, and a denser fiber distribution [(Fig. 2(a′)]. The TPEF images obtained demonstrated uniform autofluorescence with the characteristics of glandular and epithelial architecture [Fig. 2(b)]. The combined SHG and TPEF imaging results revealed that the stromal framework was well-organized and intact [Fig. 2(c)], consistent with the corresponding H&E-stained section [Fig. 2(d)].

**Fig. 2:**
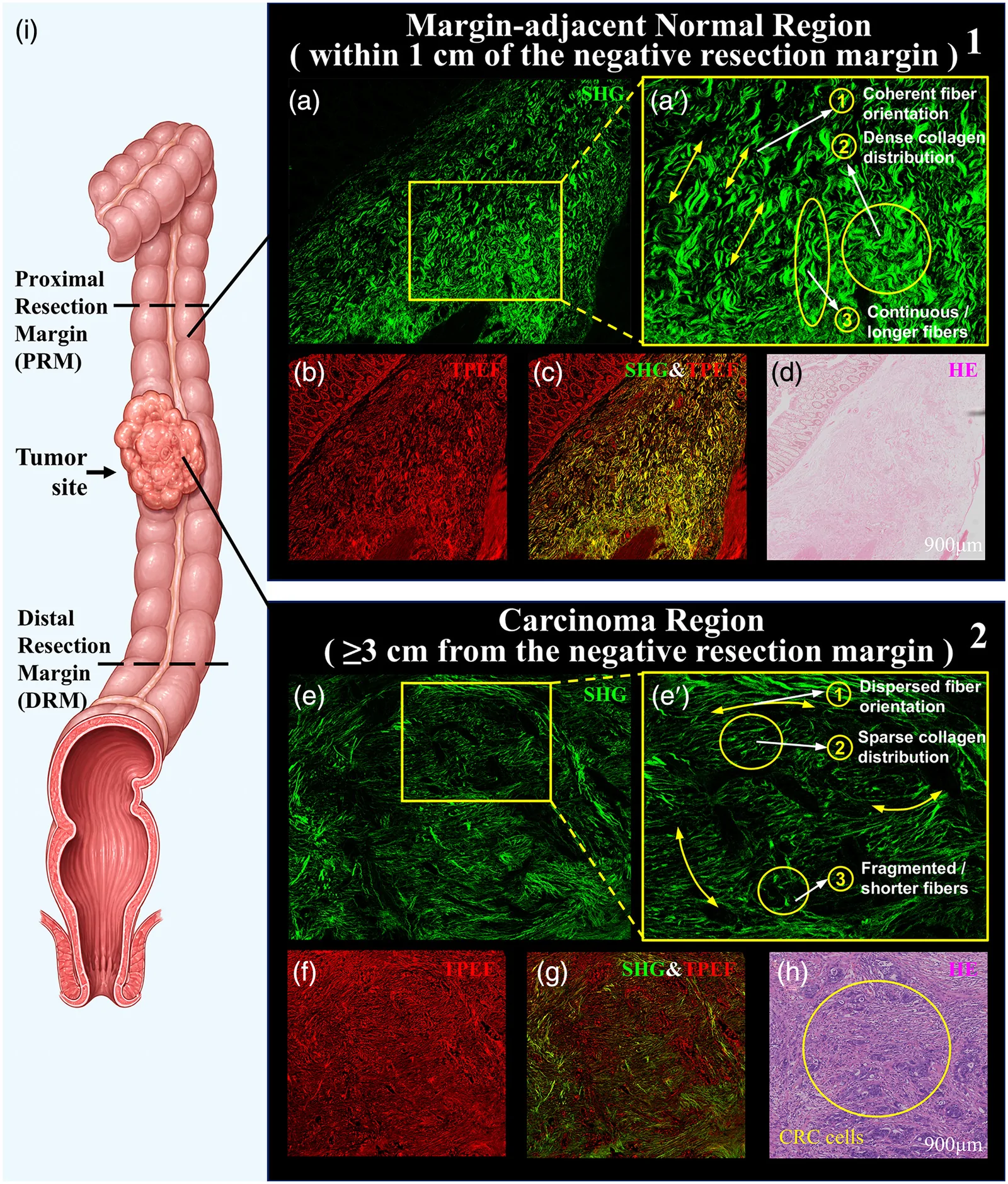
Representative multiphoton images of margin-adjacent normal and carcinoma regions. (Region 1) Margin-adjacent normal tissue within 1 cm of the negative resection margin. (a) SHG image showing collagen in green. (a′) Magnified view of the boxed ROI in (a), illustrating relatively coherent fiber orientation, longer and more continuous fibers, and higher apparent fiber density. (b) TPEF image showing endogenous autofluorescence in red. (c) Merged SHG and TPEF image. (d) Corresponding H&E-stained section. (Region 2) Carcinoma tissue located ≥3  cm from the negative resection margin. (e) SHG image. (e′) Magnified view of the boxed ROI in (e), illustrating more disorganized fiber orientation, shorter and more fragmented fibers, and lower apparent fiber density. (f) TPEF image. (g) Merged SHG and TPEF image. (h) Corresponding H&E-stained section; the yellow circle indicates an area of infiltrating colorectal carcinoma cells. (i) Schematic illustration of the tissue sampling locations relative to the proximal resection margin (PRM), distal resection margin (DRM), and tumor site. Scale bars in (d) and (h): 900  μm.

In contrast, carcinoma tissue located ≥3  cm from the negative resection margin (Fig. 2, Region 2) showed severe architectural disorganization. SHG imaging revealed reduced and heterogeneous collagen signals with evident fiber fragmentation and disorganization [Fig. 2(e)]. The magnified ROI showed a more disorganized fiber orientation, shorter and more fragmented collagen fibers, and a lower apparent fiber density [(Fig. 2(e′)]. Using TPEF imaging, we observed marked cellular atypia and disorganized glandular structure [Fig. 2(f)]. Merged visualization revealed disruption of the stromal collagen network [Fig. 2(g)], with the attenuation signal observed at the invasive tumor periphery. Label-free MPM findings showed qualitative spatial correspondence with H&E histology, confirming the diagnosis of colorectal adenocarcinoma. The area showing tumor cell infiltration is indicated in Fig. 2(h). The tissue sampling locations relative to the PRM, DRM, and tumor site are schematically illustrated in Fig. 2(i). MPM can reveal the presence of coherent collagen networks in margin-adjacent normal tissue and fragmented or disordered collagen architecture in carcinoma tissue. There is a biological significance to the differences in collagen structure. The enlarged SHG views provide qualitative visual examples of the collagen architectural differences between the two tissue regions and correspond to several quantified morphological features, particularly fiber orientation, average fiber length, and fiber density.

### 3.2 Performance Limitations of a Feature-Engineered LASSO Model for Margin Assessment

To assess the diagnostic value of collagen characteristics extracted from SHG imaging for surgical margin classification, a traditional ML baseline was constructed using LASSO for handcrafted feature selection and LR for classification (LASSO-LR). Figure 3(a) illustrates how the feature coefficients (β) change in response to an increase in the regularization parameter (λ) on a logarithmic scale. LASSO tends to induce sparsity because the coefficients are systematically driven to zero.

**Fig. 3:**
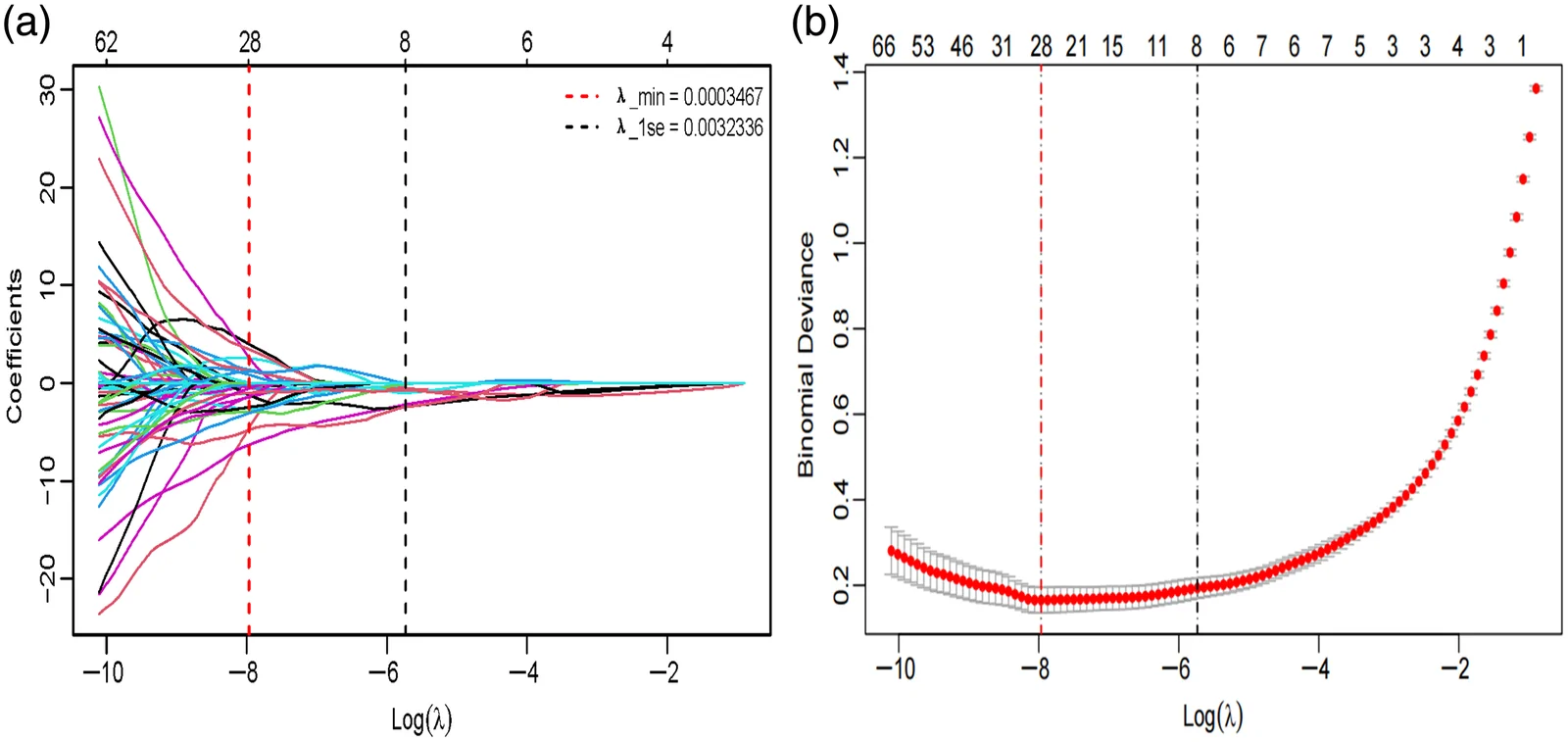
Feature selection using LASSO regression: (a) coefficient path of the LASSO regression model across different values of log(λ), where λ represents the regularization parameter; (b) cross-validation curve for selecting the optimal λ in the LASSO regression model. Binomial deviance was used to evaluate the model performance, and the vertical dashed lines indicate the optimal λ values selected by cross-validation.

The binomial deviance against λ is shown in Fig. 3(b). The red dots signify the average deviance for each of the different cross-validation folds, whereas the grey error bars represent the standard errors. Smaller error bars indicate lower variability in cross-validated deviance across folds. The left dashed line represents λ_min=0.0003467, which is the point of minimum deviance that retained 28 features. λ_1se=0.0032336 selected a more parsimonious eight-feature model whose cross-validated deviance was within one standard error of the minimum. An intercept term with a linear predictor was created using the features selected for λ_1se. The linear predictor was then transformed into a predicted probability of CRC using the sigmoid function. Values exceeding the threshold were classified as CRC tissue, whereas those below the threshold were classified as margin-adjacent normal tissue.

This LASSO-LR model proved to be a good discriminator for the target of interest, with an ROC AUC of 0.886 (95% CI, 0.854 to 0.918) [Fig. 4(a)]. One important observation is that the ROC curve rises steeply in the low false-positive-rate region, with the true positive rate rapidly reaching ∼0.7. This suggests that the model can identify a substantial proportion of CRC samples while maintaining a low false-positive rate, indicating its potential utility under high-specificity conditions. The confusion matrix [Fig. 4(b)] corresponded to an overall accuracy of 81.0% (n=389). The specificity was 86.9% (119/137), and the sensitivity for CRC classification was 77.8% (196/252). Nevertheless, 56 (22.2%) CRC images were misclassified as margin-adjacent normal tissue. Given the false-negative rate with a large possible effect and clinical significance, the intraoperative use of these tests raises particular concerns. The presence of unidentified foci of stromal invasion at the resection margin suggests incomplete tumor resection (non-R0 status) and a risk of recurrence.

**Fig. 4:**
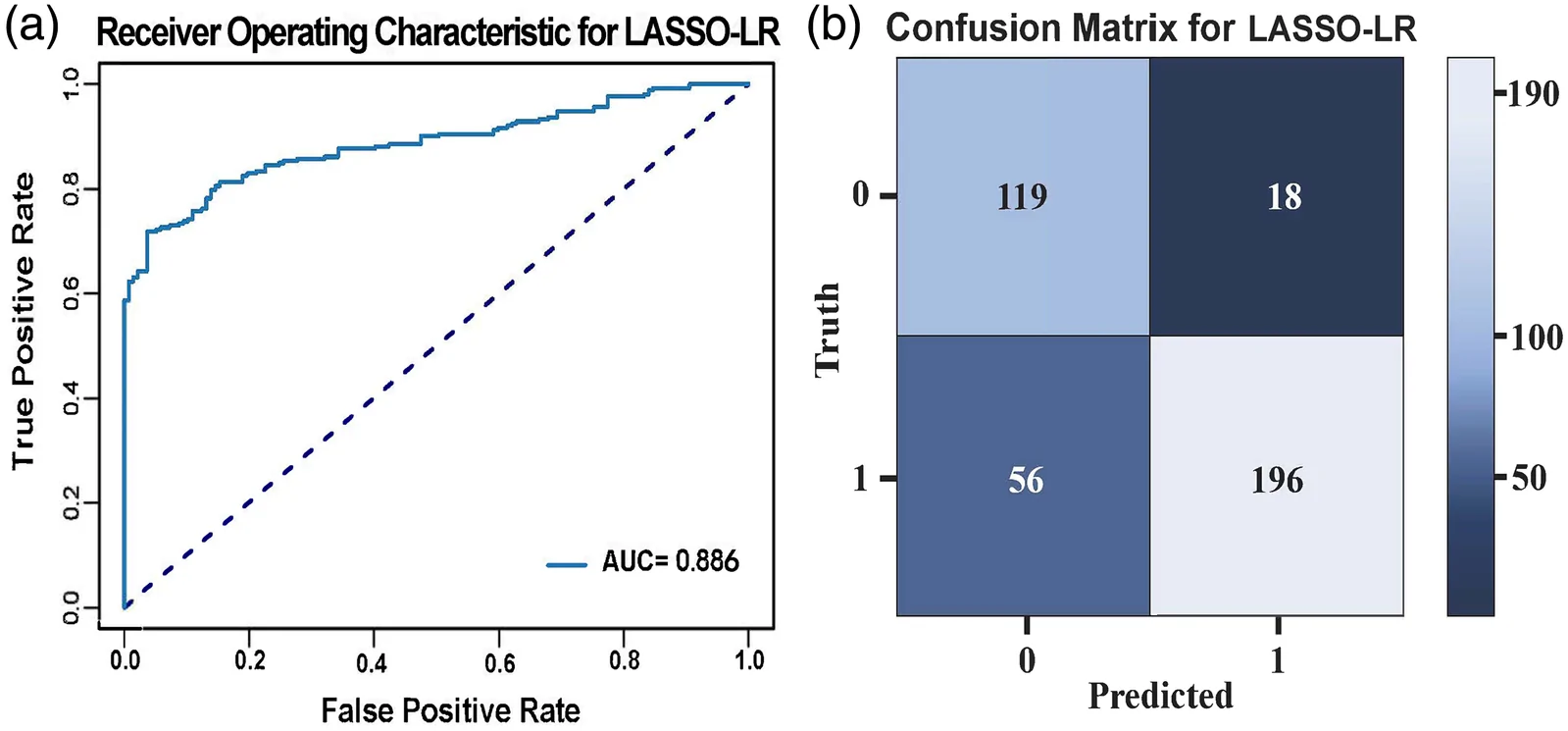
Performance evaluation of the LASSO-LR model. (a) Receiver operating characteristic (ROC) curve of the LASSO-LR model. (b) Confusion matrix of the LASSO-LR model. Class 0: margin-adjacent normal tissues. Class 1: CRC tissues.

### 3.3 Deep Learning Models and Hybrid Fusion for Margin Assessment

All classification analyses were performed using SHG images. ResNet-50, VGG-16, MobileNet-v2, and PVTv2-b5 were evaluated as image-only models, whereas PVT-Enhanced additionally incorporated the 142 quantitative collagen features extracted from SHG images. The three CNNs discriminated between margin-adjacent normal tissue (Class 0) and CRC tissue (Class 1), with ROC AUCs ranging from 0.855 to 0.870 [Figs. 5(a)–5(c)]. Their discrimination was comparable to that of the LASSO-LR baseline. These results indicate that image-only CNNs can capture discriminative collagen-architecture patterns from SHG images. The confusion matrices revealed different sensitivity–specificity trade-offs among the three CNNs [Figs. 5(d)–5(f)]. ResNet-50 showed the highest specificity (92.7%), whereas MobileNet-v2 showed the highest sensitivity (76.2%).

**Fig. 5:**
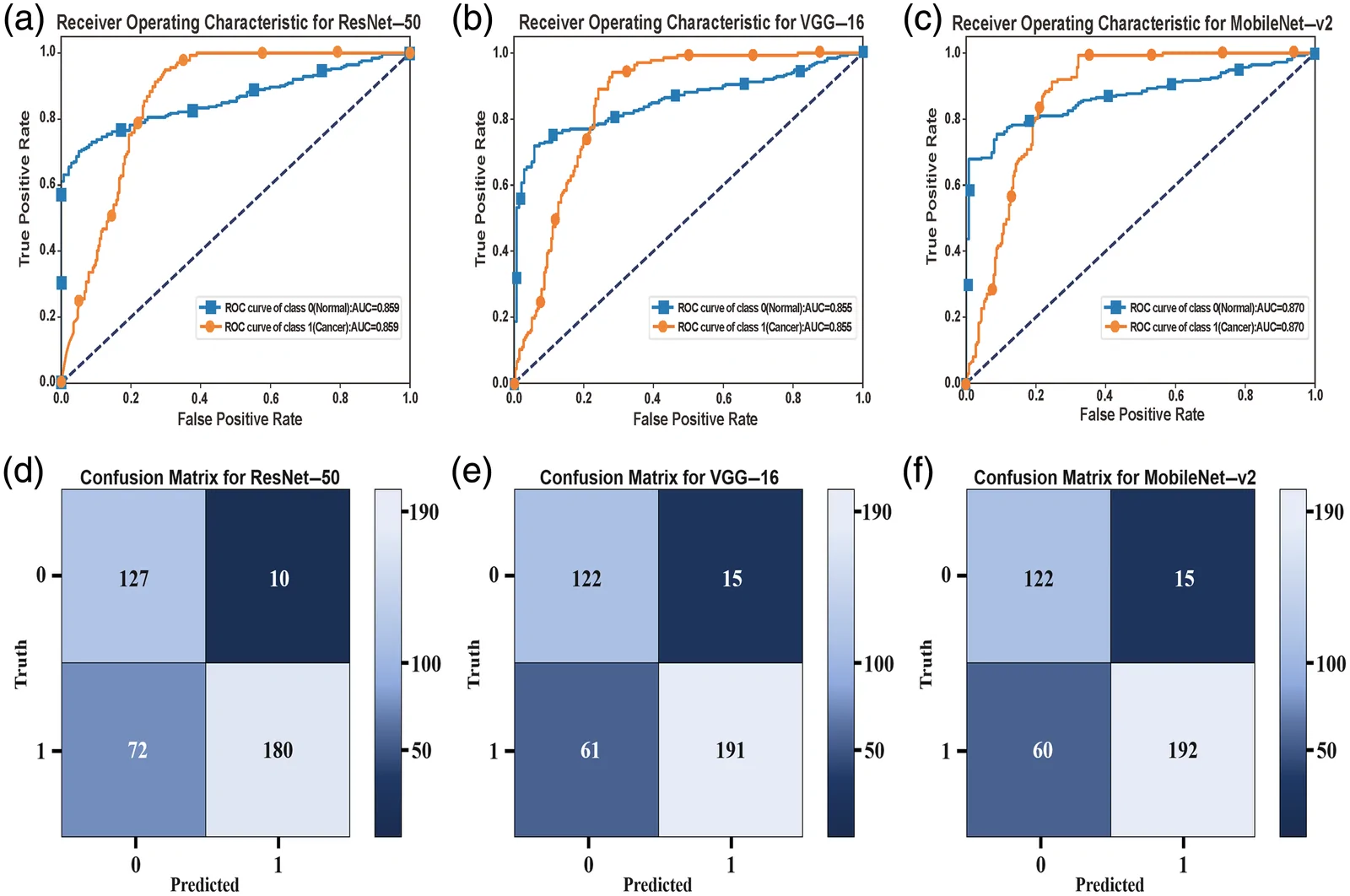
Performance evaluation of three conventional CNN models. (a–c) Receiver operating characteristic (ROC) curves of ResNet-50, VGG-16, and MobileNet-v2, respectively. (d–f) Confusion matrices of ResNet-50, VGG-16, and MobileNet-v2, respectively. Class 0: margin-adjacent normal tissues. Class 1: CRC tissues.

To complement the local feature extraction of CNNs with modeling of long-range spatial dependencies, we evaluated PVTv2-b5, which uses hierarchical self-attention to capture multi-scale contextual information from SHG images. The baseline PVTv2-b5 model achieved the highest AUC among the image-only models [0.898; Fig. 6(a)]. By integrating 142 handcrafted collagen features with the PVTv2-b5 image features, we developed the PVT-Enhanced model. An ablation analysis was performed to evaluate the contribution of the 142 handcrafted collagen features by comparing PVT-Enhanced with the image-only PVTv2-b5 baseline. Overall, PVT-Enhanced achieved the best classification performance among the evaluated feature-only, image-only, and feature–image fusion approaches. Compared with PVTv2-b5 alone, the addition of handcrafted collagen features increased accuracy from 82.8% to 86.9% and AUC from 0.898 to 0.923. The difference in AUC remained statistically significant after Holm correction (adjusted P=0.039; Table 3). The PVT-Enhanced confusion matrix showed five false-positive predictions among the 137 margin-adjacent normal images in the held-out validation cohort, corresponding to a specificity of 96.4% [Fig. 6(d); Table 3].

**Fig. 6:**
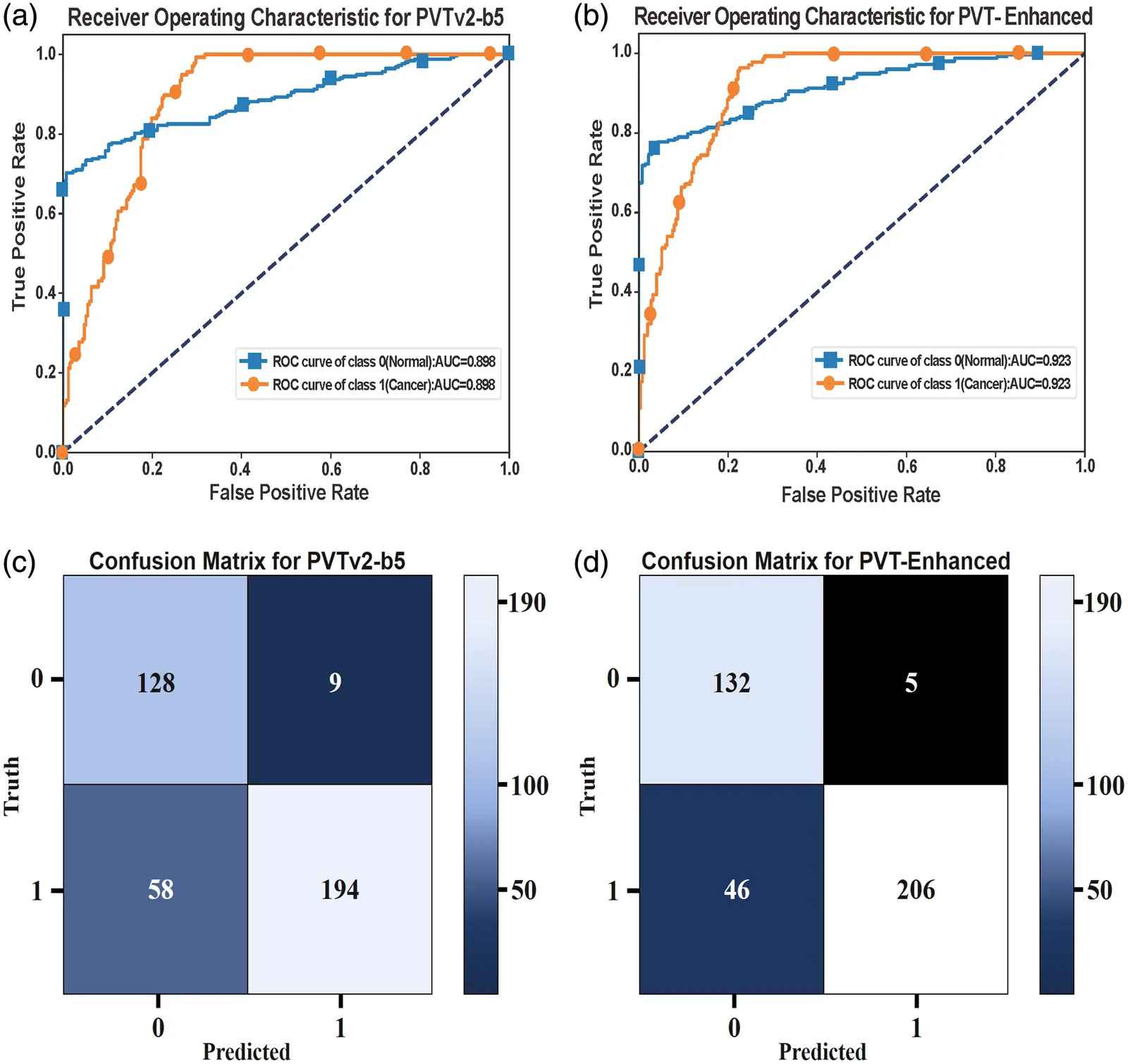
Performance evaluation of PVTv2-b5 and PVT-Enhanced classification of CRC and margin-adjacent normal tissue. (a, b) ROC curves of the PVTv2-b5 and the PVT-Enhanced, respectively. The PVT-Enhanced was constructed by integrating the PVTv2-b5 deep features with 142 handcrafted collagen features. (c, d) Confusion matrices of the PVTv2-b5 and PVT-Enhanced, respectively. Class 0 denotes margin-adjacent normal tissues, whereas Class 1 denotes CRC tissues. The PVTv2-b5 achieved TP=194, TN=128, FN=58, and FP=9, while the PVT-Enhanced achieved TP=206, TN=132, FN=46, and FP=5.

**Table 3:** Diagnostic performance of feature-only, image-only, and feature-enhanced models for CRC margin assessment on the held-out validation cohort.

Model	Input	AUC (95% CI)	Acc. (95% CI)	Sens. (95% CI)	Spec. (95% CI)	PPV (95% CI)	F1 (95% CI)	Adj. P
LASSO-LR	Features only	0.886 (0.854 to 0.918)	81.0% (76.8 to 84.6)	77.8% (72.2 to 82.5)	86.9% (80.2 to 91.5)	91.6% (87.1 to 94.6)	84.1% (80.5 to 87.6)	<0.001
ResNet-50	Image-only (CNN)	0.859 (0.824 to 0.895)	78.9% (74.6 to 82.7)	71.4% (65.6 to 76.7)	92.7% (87.1 to 96.0)	94.7% (90.6 to 97.1)	81.4% (77.3 to 85.3)	<0.001
VGG-16	Image-only (CNN)	0.855 (0.818 to 0.892)	80.5% (76.2 to 84.1)	75.8% (70.1 to 80.7)	89.1% (82.7 to 93.3)	92.7% (88.3 to 95.5)	83.4% (79.6 to 86.9)	<0.001
MobileNet-v2	Image-only (CNN)	0.870 (0.836 to 0.905)	80.7% (76.5 to 84.3)	76.2% (70.6 to 81.0)	89.1% (82.7 to 93.3)	92.8% (88.4 to 95.6)	83.7% (79.8 to 87.1)	<0.001
PVTv2-b5	Image-only (PVT)	0.898 (0.864 to 0.932)	82.8% (78.7 to 86.2)	77.0% (71.4 to 81.8)	93.4% (88.0 to 96.5)	95.6% (91.8 to 97.7)	85.3% (81.5 to 88.6)	0.039
**PVT-Enhanced**	**Image + features**	**0.923** **(0.897 to 0.948)**	**86.9%** **(83.2 to 89.9)**	**81.7%** **(76.5 to 86.0)**	**96.4%** **(91.7 to 98.4)**	**97.6%** **(94.6 to 99.0)**	**89.0%** **(85.8 to 91.9)**	**Ref.**

All models were evaluated on the same held-out validation cohort (n = 389; 252 CRC and 137 margin-adjacent normal images). The reported performance values are point estimates from this fixed held-out cohort. Adj. P, Holm-adjusted P value from the paired DeLong test comparing the AUC of each model with that of PVT-Enhanced; PVT-Enhanced served as the reference model. CI, confidence interval; AUC, area under the receiver operating characteristic curve; Acc., accuracy; Sens., sensitivity; Spec., specificity; PPV, positive predictive value; F1, F1 score; LASSO, least absolute shrinkage and selection operator; LR, logistic regression; CNN, convolutional neural network; PVT, pyramid vision transformer; CRC, colorectal cancer.

## 4 Discussion

This study conducted a comparative evaluation of various ML and DL algorithms that can be employed for automating the resection margin assessment of colorectal adenocarcinoma surgery using SHG collagen imaging. The results illustrate that representative deep-learning architectures have the potential to achieve high diagnostic performance, especially when coupled with biologically relevant features.

First, we built a baseline using a standard ML pipeline with LASSO regression for feature selection from 142 handcrafted collagen descriptors and logistic regression classification (LASSO-LR). Although the model has high classification performance (accuracy: 81.0%, AUC: 0.886), the approach still has important limitations. Owing to its linear nature and the fact that features are selected in a multi-step feature-engineering pipeline, LASSO-LR has limited ability to capture the complex, nonlinear tissue changes that occur with early or diffuse invasion of the tumor. Importantly, the 22.2% false-negative rate that we observed is a significant clinical weakness for intraoperative margin assessment because missed tumor involvement at the resection margin could increase the risk of residual disease (R1 resection).

To overcome these drawbacks and improve efficiency and robustness, we shifted to end-to-end learning with the aid of three representative convolutional neural networks (CNNs):^31^ ResNet-50, VGG-16, and MobileNet-v2. Their overall performance was comparable to that of the LASSO-LR model. Among the three CNNs, ResNet-50 showed the highest specificity (92.7%), whereas MobileNet-v2 achieved the highest sensitivity (76.2%). Nonetheless, the essential reliance of CNNs on local receptive fields limits their ability to model changes in global tissue architecture through the margins of tumor invasion, a disorganization of collagen that often extends beyond local convolution.^32^

To overcome this limitation, we used the PVTv2-b5 architecture, which combines local convolutional feature extraction with hierarchical global self-attention. By combining convolution-based local feature extraction with hierarchical transformer modeling, this design allows for the effective capture of long-range dependencies and multiscale structural patterns in tissue organization while maintaining a fine spatial resolution. Through our experiment involving PVTv2-b5, a considerable improvement in performance was achieved, with an AUC of 0.898, demonstrating the efficacy of global context modeling for precise tissue discrimination. In addition, we added 142 quantitative handcrafted collagen features to the deep features learned by the transformer backbone and incorporated channel–spatial attention for feature recalibration and multi-scale feature fusion. This hybrid fusion strategy combines end-to-end learned image representations with explicit quantitative collagen features to identify collagen pathology. The model achieved an AUC of 0.923 and a specificity of 96.4% in the held-out validation cohort, with a low false-positive rate for margin-adjacent normal tissue.

Our results illustrate the potential of an imaging–model co-design strategy that combines label-free MPM/SHG imaging with advanced PVT architectures for high-performance tissue classification in surgical margin assessment. The MPM/SHG technique enables visualization at sub-micron resolution without the use of exogenous contrast agents, preserving the structural signature of collagen in its native state. Consequently, artifacts arising from standard histochemical processing were avoided. The global self-attention mechanism of the PVT architecture mitigates the limited receptive-field constraint of CNNs.^32^ The model can perceive both the micro-arrangement and macro-disorganization of collagen fibers. This is important for defining the often irregular and diffusing infiltrative edges of tumor invasion. The addition of handcrafted features representing malignancy-associated collagen remodeling, including fiber alignment and collagen crosslinking,^13^^,^^33^ provided complementary quantitative information and improved classification performance. These explicit collagen descriptors provide biologically meaningful context for the fused representation. The representative SHG ROIs in Fig. 2(a′) and 2(e′) visually illustrate this remodeling through differences in fiber orientation, fiber continuity/length, and collagen distribution, providing an intuitive morphological basis for the corresponding handcrafted descriptors.

This study established a proof-of-concept framework relevant to intraoperative margin assessment. Our hybrid approach using the transformer model favors end-to-end learning of the global tissue context and employs biologically relevant collagen descriptors. Among all evaluated models, the PVT-Enhanced model achieved the best overall performance on the held-out validation cohort, with an accuracy of 86.9%, sensitivity of 81.7%, specificity of 96.4%, AUC of 0.923, and PPV of 97.6%. Notably, the high specificity of the model is of particular clinical relevance for reducing false-positive margin assessments and avoiding unnecessary removal of noncancerous tissue. These findings represent a step toward developing a clinically applicable approach that may ultimately support intraoperative margin assessment and contribute to reducing the risk of R1 resection in colorectal cancer surgery. Further validation in larger multicenter cohorts is needed, together with investigation of whether the proposed AI-based diagnostic framework can be combined with handheld or endoscopic MPM systems for real-time assessment of intact tissue margins during surgery.

## 5 Conclusion

In this study, we developed PVT-Enhanced, a feature-fusion framework that integrates hierarchical image features learned from SHG images by PVTv2-b5 with 142 handcrafted collagen features for distinguishing CRC from margin-adjacent normal tissue. The proposed approach showed potential for label-free assessment of resection margins in colorectal cancer surgery. However, the current study is limited by its single-center cohort and relatively small sample size. These findings provide a basis for further development of the proposed framework toward rapid, label-free assessment of intact tissues in surgical pathology.

## Data Availability

The data that support the findings of this study are available from the corresponding author upon reasonable request.
